# Impact of post-diagnosis weight change on survival outcomes in Black and White breast cancer patients

**DOI:** 10.1186/s13058-021-01397-9

**Published:** 2021-02-04

**Authors:** Lihua Shang, Masaya Hattori, Gini Fleming, Nora Jaskowiak, Donald Hedeker, Olufunmilayo I. Olopade, Dezheng Huo

**Affiliations:** 1 Department of Medical Oncology, Harbin Medical University Cancer Hospital, Harbin, China; 2 Department of Breast Oncology, Aichi Cancer Center, Nagoya, Japan; 3 Section of Hematology and Oncology, Department of Medicine, University of Chicago, Chicago, IL USA; 4 Department of Surgery, University of Chicago, Chicago, IL USA; 5 Department of Public Health Sciences, University of Chicago, 5841 S. Maryland Avenue, MC 2000, Chicago, IL 60637 USA

**Keywords:** Body mass index, Weight change, Breast cancer, Prognosis, Racial disparities

## Abstract

**Purpose:**

To evaluate weight change patterns over time following the diagnosis of breast cancer and to examine the association of post-diagnosis weight change and survival outcomes in Black and White patients.

**Methods:**

The study included 2888 women diagnosed with non-metastatic breast cancer in 2000–2017 in Chicago. Longitudinal repeated measures of weight and height were collected, along with a questionnaire survey including questions on body size. Multilevel mixed-effects models were used to examine changes in body mass index (BMI). Delayed entry Cox proportional hazards models were used to investigate the impacts of changing slope of BMI on survival outcomes.

**Results:**

At diagnosis, most patients were overweight or obese with a mean BMI of 27.5 kg/m^2^ and 31.5 kg/m^2^ for Blacks and Whites, respectively. Notably, about 45% of the patients had cachexia before death and substantial weight loss started about 30 months before death. In multivariable-adjusted analyses, compared to stable weight, BMI loss (> 0.5 kg/m^2^/year) showed greater than 2-fold increased risk in overall survival (hazard ratio [HR] = 2.60, 95% CI 1.88–3.59), breast cancer-specific survival (HR = 3.05, 95% CI 1.91–4.86), and disease-free survival (HR = 2.12, 95% CI 1.52–2.96). The associations were not modified by race, age at diagnosis, and pre-diagnostic weight. BMI gain (> 0.5 kg/m^2^/year) was also related to worse survival, but the effect was weak (HR = 1.60, 95% CI 1.10–2.33 for overall survival).

**Conclusion:**

BMI loss is a strong predictor of worse breast cancer outcomes. Growing prevalence of obesity may hide diagnosis of cancer cachexia, which can occur in a large proportion of breast cancer patients long before death.

## Introduction

Obesity is a common health problem in the USA with its prevalence increasing in the past few decades [1]. Obesity is associated with not only an increased risk of many cancers [2], including postmenopausal breast cancer [3], but may also impact cancer prognosis and treatment [4]. Body size before or at diagnosis and survival has been studied extensively. A recent meta-analysis reported that for a 5 kg/m^2^ increase in body mass index (BMI) before diagnosis, overall mortality and breast cancer mortality risk increased by 17% and 18%, respectively [5]. However, relatively few studies have investigated the relationship between weight change after diagnosis and survival outcomes in breast cancer patients, with heterogeneous results [5, 6]. Several studies found an association of weight loss with increased risk of mortality [6–11]. While some studies found an association of weight gain with increased risk of mortality [6, 8, 9, 12], other studies did not find an association between weight gain and survival [10, 11].

Most previous studies of weight change only assessed body weight at one time point post-diagnosis, usually within the first 2 years. Some research relied only on self-reported body weight rather than objectively measured weight. Furthermore, few previous studies of weight change included racial/ethnic minorities in the USA. It is known that obesity prevalence varies across race and ethnicity, and Black women have higher obesity rates compared with other racial/ethnic groups [1, 13]. In the US population, 57% of Black women are obese, while obesity for Hispanic women is 47%, 39% for White women and 12% for Asian American women [13]. Due to heterogeneity of disease phenotypes and treatments, several questions remain about weight change and survival for Black women in the USA with breast cancer. While it is well documented that Black women with breast cancer have higher mortality rates compared with other racial/ethnic groups in the USA [14], it remains unclear whether higher prevalence of obesity in Black women contributes to the racial disparity in breast cancer mortality [4, 15, 16]. It is also unclear whether there are racial differences in patterns of weight change after breast cancer diagnosis and if so, how these different patterns of weight change effect breast cancer survival.

To address these knowledge gaps, we used electronic health records and survey data from a large, multi-ethnic cohort of women diagnosed with breast cancer between 2000 and 2017 and treated at the University of Chicago Medicine to evaluate weight change patterns over time by race and ethnicity and to examine the effect of post-diagnosis weight change on survival outcomes. To the best of our knowledge, this is a large single institution study to examine serial weight changes using multiple measurements from electronic health records and identifies significant real world evidence for the effect of weight change on cancer mortality in Black women in the USA.

## Patients and methods

### Study population

The Chicago Multiethnic Epidemiologic Breast Cancer Cohort (ChiMEC) was initiated as a hospital-based case-control study to facilitate research on the effects of high-penetrance susceptibility genes, common genetic variants, and environmental risk factors for breast cancer [17–20]. Breast cancer cases were followed for survival, disease recurrence, and other outcomes to form the ChiMEC cohort. Patients diagnosed or treated at the University of Chicago Hospitals were ascertained through the cancer risk clinic established in 1992. In 2008, the study was expanded to recruit all breast cancer patients evaluated in the institution. Clinical, pathological, and treatment data were collected via electronic medical records. Epidemiological risk factor data were collected via a structured questionnaire. A biobank was established, collecting blood and tumor samples, including both fresh frozen and formalin-fixed, paraffin-embedded tumor blocks. Enrollment and follow-up of the patients is on-going. This analysis includes female patients who were ≥ 18 years of age at diagnosis, enrolled in the ChiMEC study between 2000 and 2017, had histologically diagnosed non-metastatic breast cancer, and available body weight and height data. The study protocol was approved by the institutional review board of the University of Chicago.

### Measurement of body weight and height

**Fig. 1:**
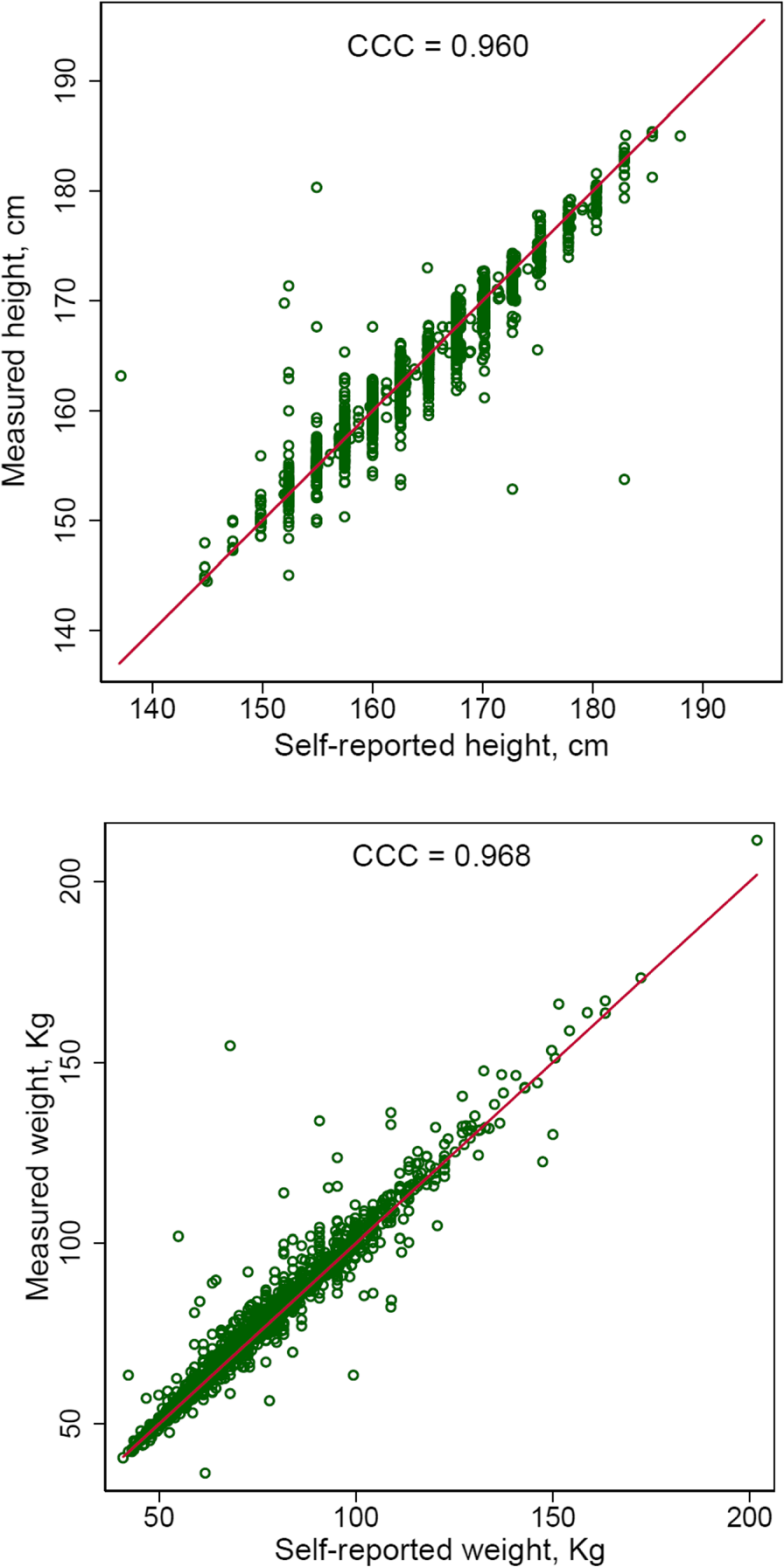
Concordance plot of measured and self-reported height and weight. CCC, Lin’s concordance correlation coefficient for agreement

Body weight was measured repeatedly and recorded in electronic health records in the hospitals. We also conducted phone or online interview survey and asked patient’s body weight and height in 19 months after diagnosis on average. In a subset of 1524 patients, both measured and self-reported weights were available on similar dates (difference between dates of measurement and interview < 6 months, median 34 days separation). Self-reported and measured weights of these patients were highly correlated, with a Lin’s concordance correlation coefficient of 0.968 (Fig. 1). On average, measured weight was 1.5 kg higher than self-reported weight. Because of this high correlation, we utilized calibrated weights at date points without measured weights but with self-reported weights; the calibrated weights were calculated from reduced major axis (RMA) regression. At date points with measured weights, we took the values directly. The self-reported and measured heights were also highly correlated with a concordance correlation coefficient of 0.960 and an average difference of 0.2 cm.

BMI was calculated as weight in kilograms divided by the square of height in meters (kg/m^2^) and categorized according to the World Health Organization definition: underweight (< 18.5 kg/m^2^), normal weight (18.5–24.9 kg/m^2^), overweight (25.0–29.9 kg/m^2^), or obese (≥ 30 kg/m^2^).

### Covariates

Demographic and lifestyle data were collected at baseline, and included race, ethnicity, gender, age at diagnosis, smoking history, and insurance status. Clinical data included date of diagnosis, tumor stage, tumor size, number of lymph nodes involved, histological type, histological grade, estrogen receptor status, progesterone receptor status, HER2 status, surgery, chemotherapy, radiotherapy, and hormonal therapy. Comorbidity status was represented as Deyo/Charlson comorbidity index [21], which was a cumulative score of 15 health conditions at date of breast cancer diagnosis.

### Survival outcomes

Patient outcomes were collected from the University of Chicago Cancer Registry and records of clinical visits, and through periodic searches of the National Death Index. Outcomes were death due to all-cause mortality, death due to breast cancer, loco-regional and distant recurrence, and second primary breast cancer. We examined three survival endpoints using delayed entry Cox proportional hazards models [10]. Time since diagnosis was used as the time scale. The entry date in the Cox model was the date of last post-diagnosis weight measurement, rather than date of diagnosis to avoid survivors’ bias. The exit dates for the three survival outcomes were specified differently. In the overall survival analysis, it was the date of death from any cause or last date of follow-up. The exit date of breast cancer-specific mortality analysis was the date of death due to breast cancer or last date of follow-up. The exit date of disease-free survival was the date of recurrence, second primary breast cancer, death or last date of follow-up, whichever came first.

### Statistical analyses

Baseline demographic and clinical factors were compared between racial/ethnic groups using chi-square tests for categorical variables and linear regression models for continuous variables. To examine factors related to post-diagnostic weight change, we fit three-level mixed-effects regression models for BMI [22]. The first-level consisted of the BMI values at each time point, which were nested within second-level time periods, and then within third-level individual patients. We specified 5 time periods: time at diagnosis, then at < 12 months, 12–24 months, 24–36 months, and 36–60 months post diagnosis. BMI at diagnosis refers to any weights measured between < 1 year before diagnosis and before any cancer therapy. We included second and third-level random intercepts and third-level random slopes in the models to account for correlation among BMIs within time periods and individuals. On average, there were 31 BMI measures per patient, but because the number of observations per patient varied largely across patients, we utilized three-level rather than two-level models to avoid undue influence from patients having many recorded observations. In addition, we modeled the shape of BMI changes according to months before death or censoring using restricted cubic spline functions in the mixed-effects model, after stratification by vital status in both White and Black women.

We used a two-step approach to examine the impact of weight change on survival outcomes. In the first step, for each subject, we fit fixed-effect linear regression models of BMI measured at least one year before the last follow-up or death (to reduce the influence of drastic weight before death). In the second step, the slopes from the linear models (BMI changes per year) were included as an independent variable in delayed entry Cox proportional hazards models. In multivariable models, we adjusted for age, race, Carlson comorbidity index, tumor stage, histologic grade, molecular subtypes, radiotherapy, hormonal therapy, and chemotherapy. To explore whether recurrence influence the association between weight change and overall survival, we modeled recurrences as a time-varying covariate in Cox model. We also conducted stratified analysis according to race, baseline BMI, and age at diagnosis. For sensitivity analyses, we estimated slopes of BMI changes using only BMI data within the first 2 years after diagnosis and calculated BMI changes between baseline and 18 months post- diagnosis. In addition to the two-step approach, we jointly modeled longitudinal and survival data using a mixed-effects model and Weibull survival model and found the results are similar [23]. As two-step approach is faster and more flexible (allowing Cox model), we did not present the Weibull model results. Statistical analyses were conducted using the STATA 15 software package (StataCorp, College Station, TX).

## Results

**Fig. 2:**
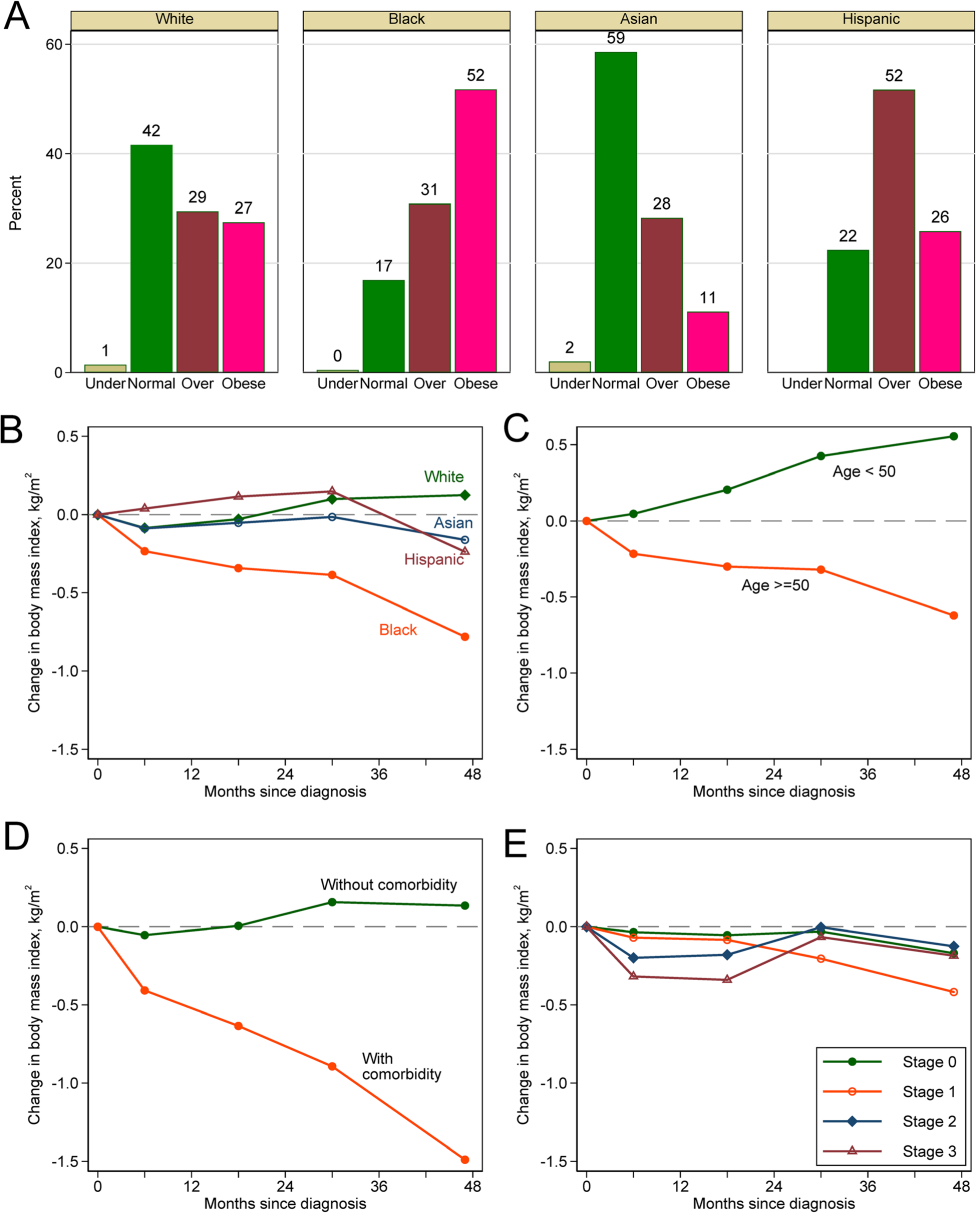
Distribution of body mass index at baseline by race/ethnicity (**a**) and changes of body mass index since breast cancer diagnosis according to race/ethnicity (**b**), age (**c**), comorbidity status (**d**), and tumor stage (**e**)

Of the 2888 breast cancer patients in the study cohort, 1575 (54%) were non-Hispanic White, 1106 (39%) Black, 126 (4%) Asian, and 81 (3%) Hispanic. Baseline demographic and clinical characteristics by racial/ethnic groups are summarized in Table 1. Compared to non-Hispanic White, Black women were older, more likely to smoke currently, receive Medicare or Medicaid benefits, and have comorbidities. As expected, Black women were more likely to have triple-negative breast cancer. The distribution of tumor stage was similar among the groups. Black women had the highest BMI at diagnosis (mean 31.5 kg/m^2^) with 30.9% being overweight and 51.8% obese, followed by Hispanic, and non-Hispanic Whites, while Asian Americans had the lowest BMI (Fig. 2a).

**Table 1:** Characteristics of 2888 patients in the Chicago multiethnic epidemiologic cohort of breast cancer study*

Characteristic	White	Black	Asian	Hispanic	*P*
*N*	1575	1106	126	81	
Age at diagnosis, *n* (%)					
< 40	147 (9.3)	86 (7.8)	17 (13.5)	15 (18.5)	< 0.001
40–49	432 (27.4)	191 (17.3)	48 (38.1)	23 (28.4)	
-59	463 (29.4)	262 (23.7)	25 (19.8)	14 (17.3)	
60–69	334 (21.2)	262 (23.7)	25 (19.8)	15 (18.5)	
70+	199 (12.6)	305 (27.6)	11 (8.7)	14 (17.3)	
*Mean (SD)*	*54.7 (12.3)*	*59.6 (13.9)*	*51.1 (12.5)*	*53.8 (14.5)*	*< 0.001*
Smoking status, *n* (%)					
Never smoker	968 (63.5)	597 (55.2)	105 (90.5)	48 (62.3)	< 0.001
Previous smoker	416 (27.3)	313 (29.0)	9 (7.8)	23 (29.9)	
Current smoker	140 (9.2)	171 (15.8)	2 (1.7)	6 (7.8)	
Insurance status, *n* (%)					
No	7 (0.4)	2 (0.2)	0 (0.0)	1 (1.2)	< 0.001
Private	1149 (73.0)	433 (39.2)	95 (75.4)	52 (64.2)	
Medicaid	37 (2.3)	166 (15.0)	4 (3.2)	5 (6.2)	
Medicare	346 (22.0)	476 (43.0)	25 (19.8)	22 (27.2)	
Other/unknown	36 (2.3)	29 (2.6)	2 (1.6)	1 (1.2)	
Carlson comorbidity index, *n* (%)					
0	1318 (83.7)	762 (68.9)	108 (85.7)	63 (77.8)	< 0.001
1	114 (7.2)	134 (12.1)	11 (8.7)	6 (7.4)	
2	115 (7.3)	132 (11.9)	4 (3.2)	11 (13.6)	
3	28 (1.8)	78 (7.1)	3 (2.4)	1 (1.2)	
Tumor stage, *n* (%)					
0	263 (16.7)	195 (17.6)	24 (19.0)	9 (11.1)	0.097
1	628 (39.9)	372 (33.6)	42 (33.3)	30 (37.0)	
2	505 (32.1)	391 (35.4)	43 (34.1)	30 (37.0)	
3	179 (11.4)	148 (13.4)	17 (13.5)	12 (14.8)	
Estrogen receptor, *n* (%)					
Negative	284 (19.2)	307 (30.3)	16 (13.6)	20 (25.3)	< 0.001
Positive	1193 (80.8)	706 (69.7)	102 (86.4)	59 (74.7)	
Progesterone receptor, *n* (%)					
Negative	458 (31.1)	436 (43.0)	29 (24.6)	26 (32.9)	< 0.001
Positive	1016 (68.9)	578 (57.0)	89 (75.4)	53 (67.1)	
Her2 status, *n* (%)					
Negative	1051 (83.7)	734 (83.1)	76 (74.5)	62 (87.3)	0.084
Positive	204 (16.3)	149 (16.9)	26 (25.5)	9 (12.7)	
Molecular subtype, *n* (%)					
ER/PR+, her2-	884 (70.4)	511 (57.9)	68 (66.7)	49 (69.0)	< 0.001
ER/PR+, her2+	128 (10.2)	90 (10.2)	18 (17.6)	6 (8.5)	
ER/PR-, her2+	76 (6.1)	58 (6.6)	8 (7.8)	3 (4.2)	
Triple negative	167 (13.3)	223 (25.3)	8 (7.8)	13 (18.3)	
Histologic grade, *n* (%)					
1	230 (15.6)	134 (12.8)	21 (17.8)	9 (11.5)	< 0.001
2	742 (50.3)	456 (43.6)	61 (51.7)	41 (52.6)	
3	504 (34.1)	456 (43.6)	36 (30.5)	28 (35.9)	
Type of surgery, *n* (%)					
No surgery	13 (0.8)	12 (1.1)	0 (0.0)	0 (0.0)	< 0.001
Breast conserving surgery	913 (58.7)	683 (65.1)	62 (49.6)	45 (57.7)	
Mastectomy	629 (40.5)	354 (33.8)	63 (50.4)	33 (42.3)	
Chemotherapy, *n* (%)					
No	881 (55.9)	586 (53.0)	71 (56.3)	39 (48.1)	0.28
Yes	694 (44.1)	520 (47.0)	55 (43.7)	42 (51.9)	
Hormonal therapy, *n* (%)					
No	495 (31.4)	462 (41.8)	27 (21.4)	28 (34.6)	< 0.001
Yes	1080 (68.6)	644 (58.2)	99 (78.6)	53 (65.4)	
Radiotherapy, *n* (%)					
No	657 (41.7)	437 (39.5)	63 (50.0)	28 (34.6)	0.074
Yes	918 (58.3)	669 (60.5)	63 (50.0)	53 (65.4)	
BMI at baseline, *n* (%)					
Underweight	19 (1.4)	4 (0.5)	2 (2.0)	0 (0.0)	< 0.001
Normal weight	548 (41.6)	143 (16.9)	58 (58.6)	13 (22.4)	
Overweight	388 (29.5)	261 (30.9)	28 (28.3)	30 (51.7)	
Obese	362 (27.5)	438 (51.8)	11 (11.1)	15 (25.9)	
*Mean (SD)*	*27.5 (6.4)*	*31.5 (7.3)*	*24.6 (4.3)*	*28.7 (6.2)*	*< 0.001*

*Number (%) are presented if not otherwise specified and numbers are not added up to total due to missing data. In particular, Her2 data were collected for few patients with stage 0 tumors

*Abbreviation*: *BMI* body mass index, *SD* standard deviation, *ER* estrogen receptor, *PR* progesterone receptor

As shown in Fig. 2b–e, Black patients had significant weight loss after 6 months, 18 months, 30 months, and 48 months from diagnosis (0.84 kg/m^2^ reduction), while body weight did not change substantively in other racial/ethnic groups. Patients younger than 50 gained weight after diagnosis, while older patients lost weight. Patients with at least one comorbidity lost substantial weight after diagnosis. In the multivariable analysis using a mixed-effects linear model, we found that Black patients had higher BMI at diagnosis but had significant weight loss after cancer diagnosis (0.47 kg/m^2^/year reduction) compared with Whites (Table 2). Older patients or patients with comorbidities had higher BMI at diagnosis but had significantly more weight loss after diagnosis, compared with younger patients or those without comorbidities, respectively. Patients diagnosed with different tumor stages had similar baseline weight but those with advanced stage had larger weight loss in a long run. At baseline, patients with or without chemotherapy had similar body weight; at months 6 and 18, patients receiving chemotherapy had more weight loss than patients without chemotherapy, but at month 48 after diagnosis, patients receiving chemotherapy had slightly less weight loss. We also found ex-smokers at diagnosis had higher baseline BMI and lost more weight during follow-up.

**Table 2:** Multivariable mixed effect linear model of body mass index among breast cancer patients

	BMI at baseline, kg/m^2^		Slope over time in BMI, kg/m^2^/year	
	Mean (SE)*	*P*	Mean (SE)*	*P*
Race/ethnicity				
White	27.85 (0.17)	Ref.	− 0.07 (0.02)	Ref.
Black	31.88 (0.20)	< 0.001	− 0.47 (0.02)	< 0.001
Asian	25.39 (0.60)	< 0.001	− 0.30 (0.05)	< 0.001
Hispanic	29.54 (0.73)	0.023	− 0.08 (0.07)	0.94
Age at diagnosis				
< 50	28.75 (0.22)	Ref.	− 0.16 (0.02)	Ref.
≥ 50	30.26 (0.16)	< 0.001	− 0.33 (0.01)	< 0.001
Carlson comorbidity index at diagnosis				
0	29.30 (0.15)	Ref.	− 0.19 (0.01)	Ref.
1+	30.96 (0.26)	< 0.001	− 0.50 (0.02)	< 0.001
Tumor stage				
0	30.09 (0.34)	0.064	− 0.14 (0.03)	0.82
1	29.42 (0.22)	Ref.	− 0.15 (0.02)	Ref.
2	29.71 (0.21)	0.35	− 0.30 (0.02)	< 0.001
3	30.48 (0.36)	0.016	− 0.59 (0.03)	< 0.001
Chemotherapy				
No	29.48 (0.21)	Ref.	− 0.32 (0.02)	Ref.
Yes	30.00 (0.20)	0.10	− 0.25 (0.02)	0.009
Smoking status at diagnosis				
Never	29.56 (0.16)	Ref.	− 0.26 (0.01)	Ref.
Previous	30.44 (0.24)	0.002	− 0.36 (0.02)	< 0.001
Current	29.25 (0.36)	0.43	− 0.20 (0.03)	0.11

*Multivariable fitted mean body mass index (BMI) and standard errors (SE) as well as the fitted mean slope of BMI over time are presented. All the variables in the table were included in the multivariable model

**Fig. 3:**
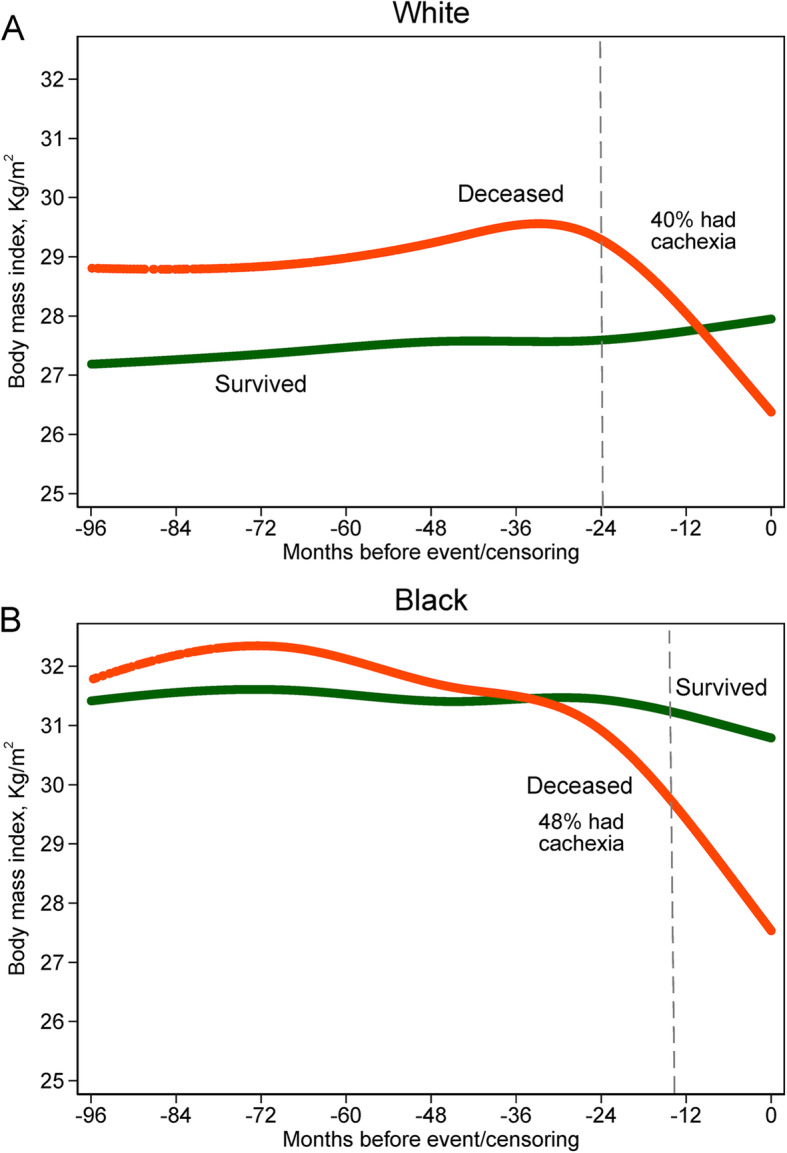
Fitted body mass index in mixed effect linear model according to months before death (for deceased patients) or last follow-up (for alive patients) among white (**a**) and black (**b**) breast cancer patients. Gray dash lines indicate median months from recurrence to death (24 months for whites and 13 months for blacks). Cachexia was defined as weight loss > 5% within 6 months

After a median follow-up time of 6.4 years, 387 patients died (190 deaths due to breast cancer) and 265 patients had a breast cancer recurrence. We modeled the shape of BMI changes according to months before death or censoring stratified by vital status in both White and Black women (Fig. 3). Among breast cancer survivors, BMI was almost constant during the follow-up years in both White and Black women. In contrast, for patients who died, there were large BMI losses starting about 30 months before death with the rapid BMI reduction observed in both Whites and Blacks (3.2 and 3.8 kg/m^2^ reduction, respectively). These BMI reductions were equivalent to 8.8 and 10.3 kg absolute weight loss for an average person in our cohort. Using a consensus definition of cancer cachexia [24], about 45% of the patients who were deceased had cachexia (40% in Whites and 48% in Blacks). The median time from recurrence to death was 24 months and 13 months for Whites and Blacks who were deceased, respectively. For patients who had distant metastasis and died later, 19% had cachexia before the recurrence.

Being obese or overweight at diagnosis was significantly associated with higher risk of all-cause mortality, breast cancer-specific mortality, and disease recurrence in the univariate analysis. However, after adjusting for age, race, Carlson comorbidity index, tumor stage, histologic grade, estrogen receptor, progesterone receptor, Her2, radiotherapy, hormonal therapy, and chemotherapy, BMI at baseline was no longer statistically associated with survival outcomes (Table 3).

**Table 3:** Prognostic values of baseline body mass index at diagnosis: Cox models

	Univariate analysis		Multivariable analysis*	
	HR (95% CI)	*P*	HR (95% CI)	*P*
**Overall survival**				
Categorical BMI				
Overweight vs. normal	1.65 (1.18–2.31)	0.004	1.23 (0.85–1.79)	0.27
Obese vs. normal	1.85 (1.34–2.55)	< 0.001	1.15 (0.79–1.65)	0.47
Continuous BMI, per 5 kg/m^2^	1.18 (1.10–1.28)	< 0.001	1.09 (0.99–1.20)	0.073
**Breast cancer-specific survival**				
Categorical BMI				
Overweight vs. normal	1.86 (1.15–3.03)	0.012	1.72 (1.01–2.93)	0.045
Obese vs. normal	1.74 (1.08–2.81)	0.023	1.36 (0.79–2.33)	0.27
Continuous BMI, per 5 kg/m^2^	1.11 (0.99–1.25)	0.079	1.01 (0.87–1.16)	0.92
**Disease-free survival**				
Categorical BMI				
Overweight vs. normal	1.65 (1.25–2.18)	< 0.001	1.30 (0.96–1.76)	0.094
Obese vs. normal	1.67 (1.28–2.19)	< 0.001	1.09 (0.80–1.48)	0.59
Continuous BMI, per 5 kg/m^2^	1.15 (1.08–1.23)	< 0.001	1.04 (0.96–1.13)	0.37

*Adjusted for age, race, Carlson comorbidity index, tumor stage, histologic grade, estrogen receptor, progesterone receptor, Her2, radiotherapy, hormonal therapy, and chemotherapy in delayed entry Cox models

*Abbreviations*: *BMI* body mass index, *HR* hazard ratio, *CI* confidence intervals

**Fig. 4:**
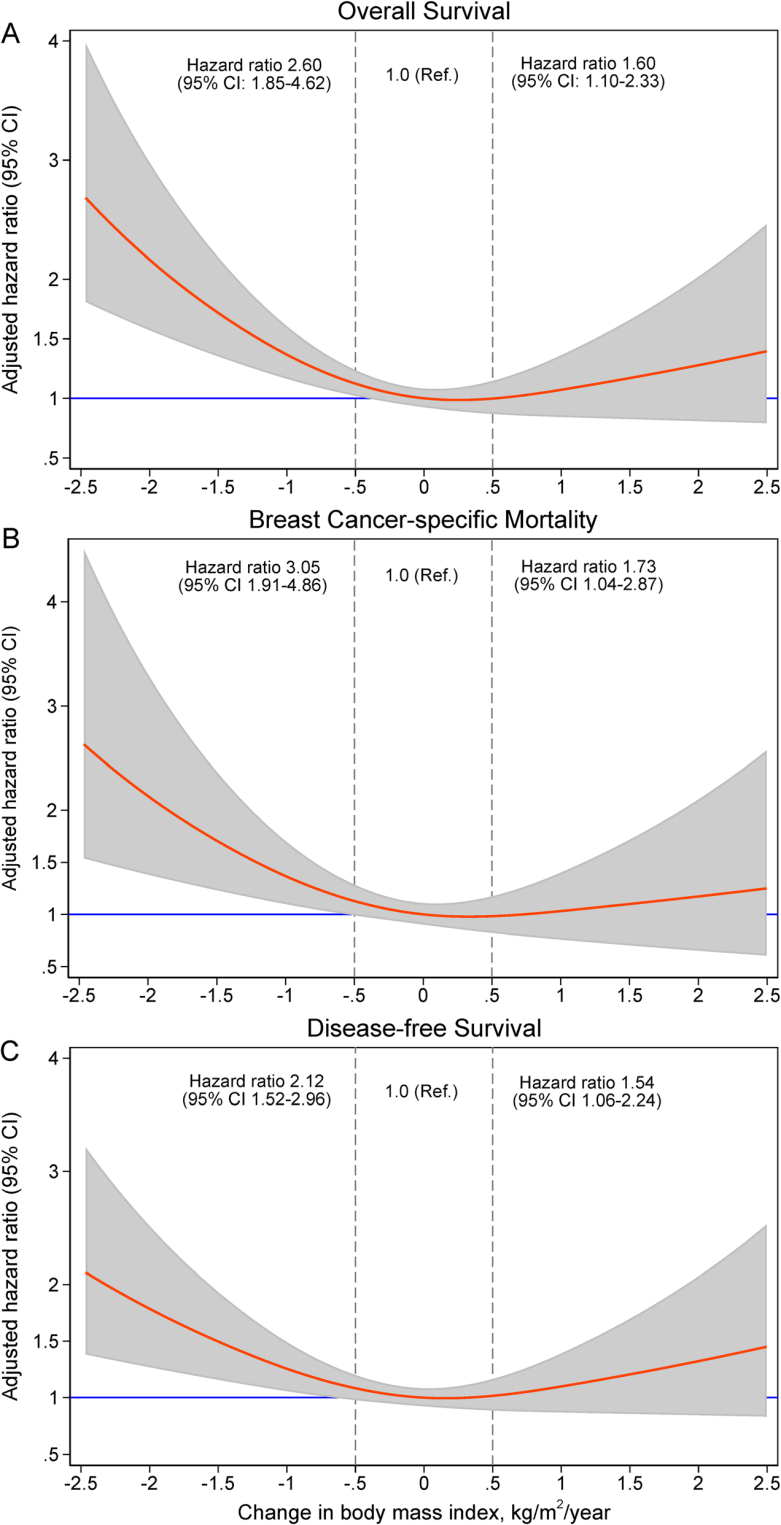
Relationship between changing slope of BMI over time (kg/m^2^/year) and three clinical outcomes: overall survival (**a**), breast cancer-specific survival (**b**), and disease-free survival (**c**). The curves were estimated from delayed entry Cox models of continuous BMI slopes fitted using restricted cubic spline, while the hazard ratios and 95% confidence intervals (CI) were estimated from Cox models that fit categorical BMI changing slopes with stable category as the reference category (BMI change less than 0.5 kg/m^2^/year)

Table 4 shows that after adjustment for multiple clinical and pathological factors, compared to stable weight, BMI loss (> 0.5 kg/m^2^/year) was associated with a higher risk of all-cause mortality (hazard ratio [HR] = 2.60, 95% CI 1.88–3.59), breast cancer-specific mortality (HR = 3.05, 95% CI 1.91–4.86), and disease-free survival (HR = 2.12, 95% CI 1.52–2.96). BMI gain (> 0.5 kg/m^2^/year) was also associated with worse survival outcomes compared with stable weight (HR = 1.60, 95% CI 1.10–2.33 for overall survival; HR = 1.73, 95% CI 1.04–2.87 for breast cancer-specific survival, and HR = 1.54, 95% CI 1.06–2.24 for disease-free survival). A slope of 0.5 kg/m^2^/year is equivalent to 3% weight change over 18 months for women with BMI of 25 kg/m^2^ (i.e., 5′5 height and 150 pounds weight). There was a U-shaped relationship between change in BMI and overall survival (Fig. 4, p-for-nonlinearity < 0.001). The larger the weight loss, the higher the risk of all-cause mortality, and BMI gain was also associated with gradual increased risk of death. Similar U-shaped relationships were also observed for breast cancer-specific survival and disease-free survival.

**Table 4:** Associations between changing slope in body mass index and clinical outcomes: multivariable Cox models*

	Overall survival		Breast cancer-specific survival		Disease-free survival	
	HR (95% CI)	*P*	HR (95% CI)	*P*	HR (95% CI)	*P*
**Changing slope in BMI**						
**Loss > 0.5 kg/m**^**2**^**/year**	**2.60 (1.88–3.59)**	**< 0.001**	**3.05 (1.91–4.86)**	**< 0.001**	**2.12 (1.52–2.96)**	**< 0.001**
**Stable (≤ 0.5 kg/m**^**2**^**/year)**	**1.0 (ref.)**		**1.0 (ref.)**		**1.0 (ref.)**	
**Gain > 0.5 kg/m**^**2**^**/year**	**1.60 (1.10–2.33)**	**0.014**	**1.73 (1.04–2.87)**	**0.035**	**1.54 (1.06–2.24)**	**0.024**
Race/ethnicity						
Black vs. White	1.98 (1.49–2.64)	< 0.001	2.06 (1.40–3.04)	< 0.001	2.02 (1.51–2.69)	< 0.001
Asian vs. White	1.38 (0.59–3.18)	0.46	1.92 (0.75–4.90)	0.18	1.21 (0.49–3.03)	0.68
Hispanic vs. White	0.32 (0.08–1.30)	0.11	0.59 (0.14–2.45)	0.47	0.31 (0.08–1.28)	0.11
Age per 10 years	1.33 (1.19–1.49)	< 0.001	1.16 (1.01–1.34)	0.036	1.35 (1.20–1.51)	< 0.001
Carlson comorbidity index						
1 vs. 0	1.28 (0.84–1.94)	0.26	1.41 (0.84–2.38)	0.20	1.35 (0.88–2.09)	0.17
2 vs. 0	2.62 (1.82–3.76)	< 0.001	1.28 (0.70–2.35)	0.43	2.87 (1.99–4.13)	< 0.001
3 vs. 0	3.36 (2.17–5.18)	< 0.001	1.05 (0.43–2.56)	0.91	3.48 (2.24–5.41)	< 0.001
Tumor stage						
0 vs. 1	0.67 (0.16–2.82)	0.59	0.0**		0.68 (0.16–2.87)	0.60
2 vs. 1	1.34 (0.96–1.86)	0.087	2.42 (1.43–4.08)	0.001	1.40 (1.00–1.97)	0.048
3 vs. 1	2.71 (1.83–4.01)	< 0.001	5.92 (3.35–10.48)	< 0.001	2.46 (1.63–3.70)	< 0.001
Histologic grade						
2 vs. 1	1.48 (0.86–2.54)	0.16	4.41 (1.06–18.41)	0.042	1.43 (0.85–2.39)	0.18
3 vs. 1	1.76 (0.97–3.17)	0.06	7.32 (1.70–31.54)	0.008	1.60 (0.91–2.84)	0.11
Molecular subtype						
ER/PR+, her2+ vs. ER/PR+/her2−	0.85 (0.52–1.40)	0.53	0.53 (0.26–1.10)	0.088	0.80 (0.48–1.33)	0.38
ER/PR−, her2+ vs. ER/PR+/her2−	0.86 (0.40–1.84)	0.70	0.93 (0.36–2.38)	0.88	0.69 (0.31–1.55)	0.37
Triple negative vs. ER/PR+/her2−	1.19 (0.66–2.14)	0.56	1.28 (0.60–2.71)	0.53	0.95 (0.52–1.74)	0.87
Radiotherapy	0.86 (0.65–1.14)	0.30	0.70 (0.48–1.02)	0.063	1.02 (0.76–1.36)	0.91
Hormonal therapy	0.93 (0.56–1.54)	0.78	0.96 (0.48–1.92)	0.91	0.96 (0.57–1.61)	0.88
Chemotherapy	1.10 (0.75–1.62)	0.62	0.91 (0.52–1.57)	0.73	1.25 (0.85–1.85)	0.26

*All variables in the table are included in the three multivariable Cox models

**No DCIS patents died from breast cancer

*Abbreviation*: *HR* hazard ratio, *CI* confidence intervals, *BMI* body mass index, *ER* estrogen receptor, *PR* progesterone receptor

In addition, we found that age was strongly associated with overall survival and disease-free survival and moderately associated with breast cancer-specific survival (Table 4). There was a monotonic strong association between higher comorbidity index with worse outcomes in overall survival and disease-free survival, but no association between comorbidity and breast cancer-specific survival. Tumor stage was strongly associated with all three survival outcomes, in particular with breast cancer-specific survival (HR = 5.92, 95% CI 3.35–10.48, comparing stage 3 vs. stage 1). Black women had about a 2-fold increased risk of the three survival outcomes compared with White women (Table 4). Consistent with a previous clinical trial-based study [25], we also found that the racial difference in overall survival was stronger among non-obese patients (HR = 2.22, 95% CI 1.48–3.33) than among obese patients (HR = 1.28, 95% CI 0.82–1.98).

We also included recurrence status as a time-varying covariate in the Cox model of overall survival and found that relationship between weight change and overall survival remained: compared to stable weight, BMI loss (> 0.5 kg/m^2^/year) was associated with a higher risk of all-cause mortality (HR = 2.55, 95% CI 1.84–3.53); BMI gain (> 0.5 kg/m^2^/year) was also associated with worse survival compared with stable weight (HR = 1.62, 95% CI 1.10–2.39). As expected, recurrence status strongly predicted overall survival (HR = 13.09, 95% CI 9.76–17.56).

In the stratified analysis of changing slope of BMI and survival outcomes, we found the U-shaped relationships were generally preserved for White and Black patients (Table 5): both weight gain and loss after diagnosis were associated with increased risk of the three outcomes. Although the effects were seemingly stronger in Blacks than in Whites, the tests for interaction were not statistically significant. The effects of BMI change on survival outcomes seemed to be limited to those women who were overweight/obese at diagnosis, but the test for interaction were not significant. BMI loss was consistently associated with worse survival outcomes regardless of the age at diagnosis.

**Table 5:** Associations between changing slope in body mass index and clinical outcomes: subgroup analysis

	Overall survival			Breast cancer-specific survival			Disease-free survival		
	Events/persons	HR (95% CI)*	*P*	Events/persons	HR (95% CI)*	*P*	Events/persons	HR (95% CI)*	*P*
**In Whites**									
Loss > 0.5 kg/m^2^/year	40/285	2.22 (1.31–3.75)	0.003	20/285	1.75 (0.86–3.55)	0.12	40/266	2.04 (1.21–3.44)	0.008
Stable (≤ 0.5 kg/m^2^/year)	30/744	1.0 (ref.)		18/744	1.0 (ref.)		31/706	1.0 (ref.)	
Gain > 0.5 kg/m^2^/year	25/372	1.41 (0.79–2.51)	0.25	15/372	1.02 (0.47–2.19)	0.96	22/348	1.33 (0.75–2.38)	0.33
**In Blacks**									
Loss > 0.5 kg/m^2^/year	93/280	2.80 (1.86–4.22)	< 0.001	46/280	4.27 (2.22–8.21)	< 0.001	83/254	2.29 (1.50–3.49)	< 0.001
Stable (≤ 0.5 kg/m^2^/year)	42/437	1.0 (ref.)		14/437	1.0 (ref.)		46/409	1.0 (ref.)	
Gain > 0.5 kg/m^2^/year	36/219	1.72 (1.05–2.84)	0.033	23/219	2.48 (1.21–5.08)	0.013	37/205	1.78 (1.08–2.92)	0.023
*Test for interaction*			*0.77*			*0.14*			*0.76*
**In women with normal weight at diagnosis**									
Loss > 0.5 kg/m^2^/year	15/77	1.92 (0.91–4.05)	0.086	8/77	2.73 (0.90–8.31)	0.077	15/74	1.91 (0.87–4.16)	0.11
Stable (≤ 0.5 kg/m^2^/year)	22/445	1.0 (ref.)		10/445	1.0 (ref.)		18/427	1.0 (ref.)	
Gain > 0.5 kg/m^2^/year	10/182	0.99 (0.45–2.16)	0.97	6/182	1.33 (0.44–4.03)	0.61	11/176	1.16 (0.53–2.55)	0.71
**In women with overweight/obese at diagnosis**									
Loss > 0.5 kg/m^2^/year	85/414	3.07 (1.96–4.80)	< 0.001	46/414	3.08 (1.62–5.87)	0.001	76/389	2.21 (1.41–3.45)	< 0.001
Stable (≤ 0.5 kg/m^2^/year)	36/647	1.0 (ref.)		16/647	1.0 (ref.)		44/613	1.0 (ref.)	
Gain > 0.5 kg/m^2^/year	30/318	1.96 (1.14–3.37)	0.015	16/318	1.47 (0.69–3.12)	0.32	27/304	1.56 (0.90–2.71)	0.11
*Test for interaction*			*0.32*			*0.98*			*0.83*
**In women ages < 50**									
Loss > 0.5 kg/m^2^/year	24/135	3.94 (1.87–8.30)	< 0.001	17/135	3.40 (1.40–8.25)	0.007	21/115	4.48 (2.05–9.79)	< 0.001
Stable (≤ 0.5 kg/m^2^/year)	13/407	1.0 (ref.)		10/407	1.0 (ref.)		11/372	1.0 (ref.)	
Gain > 0.5 kg/m^2^/year	18/281	1.58 (0.74–3.39)	0.238	16/281	1.64 (0.69–3.90)	0.27	16/259	1.65 (0.74–3.67)	0.22
**In women ages ≥ 50**									
Loss > 0.5 kg/m^2^/year	113/467	2.38 (1.67–3.38)	< 0.001	52/467	2.95 (1.73–5.04)	< 0.001	104/439	1.83 (1.28–2.62)	0.001
Stable (≤ 0.5 kg/m^2^/year)	63/882	1.0 (ref.)		25/882	1.0 (ref.)		70/845	1.0 (ref.)	
Gain > 0.5 kg/m^2^/year	45/350	1.68 (1.09–2.59)	0.019	24/350	1.83 (0.98–3.43)	0.057	45/333	1.59 (1.04–2.43)	0.032
*Test for interaction*			*0.26*			*0.86*			*0.043*

*Adjusted for age, race, Carlson comorbidity index, tumor stage, histologic grade, molecular subtype, radiotherapy, hormonal therapy, and chemotherapy in delayed entry Cox models

*Abbreviations*: *BMI* body mass index, *HR* hazard ratio, *CI* confidence intervals

We further examined whether the slope of BMI change within the first 2 years after diagnosis and BMI change from diagnosis to 18 months can predict survival outcomes (Table 6). We found that weight loss within the first 2 years of diagnosis can predict increased risk of the three survival outcomes after adjustment for other prognostic factors. Using changing slope as a measure of weight loss was more powerful in risk prediction than change between two time points, reflecting more data being efficiently utilized.

**Table 6:** Prognostic values of changes in body mass index within the first 2 years since diagnosis: multivariable Cox models

	Overall survival			Breast cancer-specific survival			Disease-free survival		
	Events/persons	HR (95% CI)*	*P*	Events/persons	HR (95% CI)*	*P*	Events/persons	HR (95% CI)*	*P*
**Slope in the first 2 years after diagnosis**									
Loss > 0.5 kg/m^2^/year	113/680	1.69 (1.20–2.40)	0.003	60/680	2.00 (1.20–3.34)	0.008	129/659	1.54 (1.13–2.11)	0.007
Stable (≤ 0.5 kg/m^2^/year)	57/804	1.0 (ref.)		25/804	1.0 (ref.)		72/786	1.0 (ref.)	
Gain > 0.5 kg/m^2^/year	59/775	1.10 (0.74–1.63)	0.63	32/775	1.26 (0.72–2.22)	0.42	86/757	1.16 (0.83–1.63)	0.39
**Change from diagnosis to 18 months**									
Loss > 5%	65/356	1.60 (1.11–2.31)	0.012	38/356	1.63 (0.99–2.69)	0.053	65/346	1.33 (0.95–1.88)	0.099
Stable (≤ 5%)	82/1127	1.0 (ref.)		40/1127	1.0 (ref.)		109/1106	1.0 (ref.)	
Gain > 5%	27/311	1.14 (0.72–1.81)	0.58	13/311	0.95 (0.49–1.84)	0.89	37/306	1.05 (0.70–1.57)	0.81

*Adjusted for age, race, Carlson comorbidity index, tumor stage, histologic grade, molecular subtype, radiotherapy, hormonal therapy, and chemotherapy in delayed entry Cox models

*Abbreviations*: *HR* hazard ratio, *CI* confidence intervals

## Discussion

In this multiethnic cohort of breast cancer patients, we demonstrated a U-shaped relationship of weight change after diagnosis with all-cause mortality, breast cancer-specific mortality, and disease-free survival. We did not find this relationship significantly different across race, age at diagnosis, and pre-diagnostic weight subgroups. The associations between weight loss and survival outcomes were strong, while the associations between weight gain and survival outcomes were weak. We also identified several factors related to weight change after breast cancer diagnosis.

We found that the patterns of post-diagnosis weight change varied by race, age, comorbidity, and tumor stage. Of interest, Black women who had the highest BMI at diagnosis (> 80% obese/overweight) also had the most post-diagnosis weight loss. Patients with any comorbidities had quite large weight loss (0.5 kg/m^2^ per year) and having comorbidities can strongly predict worse overall survival but not breast cancer-specific survival. It is possible that weight loss is an indicator of unstable comorbidity conditions. Advanced stage patients actually lost more weight, which may reflect that advanced stages predict worse survival and weight loss is a forerunner of death as shown in this and previous studies [6–11]. We found patients undergoing chemotherapy lost more weight than those without chemotherapy around the time of chemotherapy administration (within 18 months), possibly due to toxicities of chemotherapy (nausea, vomiting, and diarrhea), but in long-run, they lose less weight than patients without chemotherapy. This is consistent with a meta-analysis, which documented weight gain during chemotherapy [26], although most studies in the meta-analysis examined short-term weight change while we examined long-term weight change.

By using BMI changing slope (rate of weight loss over time), we showed strong associations between weight loss and all three survival outcomes. Although different definitions of weight loss have been used across studies, our study findings are consistent with previous studies [6–11, 27], which were mostly conducted in White patients. Interestingly, one study found a stronger association between weight loss and overall mortality and breast cancer-specific mortality in Chinese compared to White patients [10]. We found somewhat stronger effects, but not statistically significantly, in Black women than in White women. Taken together, it is reasonable to conclude that post-diagnosis weight loss is associated with worse survival in breast cancer patients, though the effect size might be different across populations. We also showed that even among women who were overweight/obese at diagnosis, weight loss did not confer a survival advantage, which is consistent with previous studies [6, 9, 10]. To mimic previous studies, we also restricted the analysis to weight change within the first 2 years after diagnosis and still found that weight loss in the first 2 years was a predictor for worse survival but the effect was attenuated compared with using all available data.

The underlying reasons for the consistent, strong association between weight loss and elevated mortality are not very clear. It is possible that patients have pre-cachexia or cachexia because of cancer treatment, tumor progression (tumor-induced inflammation), or deterioration of comorbidities. Cachexia was defined as weight loss ≥ 5% over past 6 months, and traditionally it is viewed as an end-of-life condition in patients with advanced malignancies [24]. However, we found that rapid weight loss started about 30 months before death in both White and Black patients. As a benchmark, the median time from recurrence to death in those who died was 24 months for White and 13 months for Black patients, so some deceased patients (19%) had already had cachexia before distant recurrences. This alarming observation suggests that cancer cachexia can be present early during cancer progression, which warrants further investigation and attention from clinicians. Earlier diagnosis of cancer cachexia may be important to initiate multimodal management plans including nutritional support, exercise, active anticancer therapy, and even palliative care for some patients. Cachexia is a complex metabolic condition and often involves severe depletion of skeletal muscle or sarcopenia [24, 28, 29]. Sarcopenia was found to be an independent predictor of cancer outcomes [30–32]. Similar to most large observational studies, we did not collect information on intentionality of weight loss. We postulate that most weight loss is non-intentional in our study. Although intentional weight loss has been demonstrated to be feasible and safe in the short-term among breast cancer patients [33–35], we are still waiting for results from two ongoing randomized clinical trials on the effects of intentional weight loss on cancer recurrence and survival [36, 37].

With regard to post-diagnosis weight gain and survival, previous studies gave inconsistent results. Some studies found positive association between weight gain and increased risk of all-cause mortality [6, 8, 9, 12], while others did not [10, 11]. A meta-analysis reported large weight gain (≥ 10.0%) was associated with all-cause mortality (HR = 1.23), but not for moderate weight gain (5–10%: HR = 0.97) [38]. A study published after the meta-analysis failed to show the association between large weight gain and overall survival [11]. There were also inconsistences in terms of breast cancer-specific survival and/or breast cancer recurrence; some studies found association between weight gain with increased risk of breast cancer mortality [6, 8, 12], but others did not [10, 11]. Our primary analysis in the ChiMEC cohort used the rate of weight change over time (i.e., slope) rather than change between two selected time points. We found that hazard ratios for the three survival outcomes were more than 1.5 when comparing women with weight gain slope > 0.5 kg/m^2^/year to women with stable weight (weight change ≤ 0.5 kg/m^2^/year), and the strength of association was stronger in Black women than in White women, though the tests for interaction were not significant. When we restricted the analysis to weight change in the first 2 years post-diagnosis, no statistically significant associations were found between weight gain and mortality. Taken together, the association between post-diagnosis weight-gain and survival outcomes is quite weak, so it is statistically significant only for large weight gain or using a more sensitive analytical approach. It is interesting to further investigate whether weight gain after diagnosis has different impact on survival across racial/ethnic populations. There are several possible mechanisms for the association between weight gain and elevated mortality. Weight gain, most likely gain of fatness, could increase circulating estrogens, while estrogens promote the growth of estrogen-dependent breast tumors [39] Several clinical trials demonstrated that aromatase inhibitors were not fully effective for hormone receptor-positive breast cancer patients who were obese [40]. Weight gain, particularly in overweight and obese patients, could alter levels of leptin and adiponectin and induce abnormal glucose metabolism [38]. Several studies showed that diabetes and biomarkers of glucose metabolism were associated with breast cancer death [40].

The study has several strengths. It is based on a relatively large, racially diverse sample, enhancing the generalizability of the study findings. Another strength is that the study collected longitudinal repeated measures of body size after diagnosis and analyzed the data sufficiently using state-of-the-art methods. We calculated changing rate (slope) of BMI using fixed effect linear models, as opposed to previous studies that measured weight change at a single time point after diagnosis. Our method is more accurate and reliable because it utilized all available data points and accounted for timing of measurement. As shown in Table 6, BMI changing slope gave more reliable and clear-cut interpretable results.

Several limitations need to be considered. First, some weight data were self-reported. Fortunately, at time points when we have both self-reported and measured weights, we found excellent agreement, suggesting that the self-reported weights were reliable in our cohort. There was a systematic under-reporting of weight (1.5 kg on average), which may reflect a social desire of lower weight or wearing less clothes when measuring weight at home than at hospital. We did a calibration to self-reported weights so the data should be reliable. Second, we do not know if the weight loss is intentional or not, so one should be cautious when interpreting the finding on weight loss’s effects. The large weight loss before death is most likely unintentional. Intentional weight loss, especially weight loss due to exercise, could be beneficial for breast cancer survivors. Results from several ongoing trials should shed light on this question.

## Conclusions

In summary, this study of breast cancer patients demonstrates that both weight gain and loss are associated with an adverse breast cancer prognosis, and maintaining stable weight is associated with better survival outcomes. In addition, the study suggests that cancer cachexia could start as early as 30 months before death. These findings have important implications for clinical practice. Clinicians should be aware that sudden unknown weight loss, regardless of a patient’s race, initial body weight, and age, may be a sign of poor prognosis and require a more personalized approach to disease management ranging from nutritional support to active cancer therapy.

## Data Availability

The datasets used and analyzed during the current study are available from the corresponding author on reasonable request.
